# Going through phages: a computational approach to revealing the role of prophage in *Staphylococcus aureus*

**DOI:** 10.1099/acmi.0.000424

**Published:** 2023-06-16

**Authors:** Tyrome Sweet, Suzanne Sindi, Mark Sistrom

**Affiliations:** ^1^​ Department of Life and Environmental Sciences, University of California, Merced, California, USA; ^2^​ Department of Applied Mathematics, University of California, Merced, California, USA

**Keywords:** *Staphylococcus aureus*, bacteriophages, whole genome sequences, computational biology, transduction

## Abstract

Prophages have important roles in virulence, antibiotic resistance, and genome evolution in *Staphylococcus aureus *. Rapid growth in the number of sequenced *S. aureus* genomes allows for an investigation of prophage sequences at an unprecedented scale. We developed a novel computational pipeline for phage discovery and annotation. We combined PhiSpy, a phage discovery tool, with VGAS and PROKKA, genome annotation tools to detect and analyse prophage sequences in nearly 10 011 *S *. *aureus* genomes, discovering thousands of putative prophage sequences with genes encoding virulence factors and antibiotic resistance. To our knowledge, this is the first large-scale application of PhiSpy on a large-scale set of genomes (10 011 *S *. *aureus *). Determining the presence of virulence and resistance encoding genes in prophage has implications for the potential transfer of these genes/functions to other bacteria via transduction and thus can provide insight into the evolution and spread of these genes/functions between bacterial strains. While the phage we have identified may be known, these phages were not necessarily known or characterized in *S. aureus* and the clustering and comparison we did for phage based on their gene content is novel. Moreover, the reporting of these genes with the *S. aureus* genomes is novel.

## Data Summary

All supporting data, code and protocols have been provided within the article or through supplementary data files. One supplementary file are available with the online version of this article.

**Impact Statement**

Bacteriophages (phage) play key roles in bacterial evolution, governing abundance, adaptation, and diversity of bacterial communities. Temperate phage can facilitate bacterial adaptation via transduction of novel genes. This study takes advantage of the unprecedented quantity of genomic sequencing in public repositories to analyse viral genes in 10 011 Staphylococcus aureus genomes. We found 196 727 predicted prophage genome sequences, with an estimated total of 129 935 genes. We determined the function of these genes, identifying a large quantity of novel genes that benefit the host such as beta-lactamase, enterotoxins and cytotoxins. These results will inform studies of bacterial evolution and adaptation, by identifying the mechanism of horizontal transfer of genes that confer adaptive traits to bacteria, especially in the context of antibiotic resistance.

## Introduction

The ecological importance of viruses is now widely recognized, yet our limited knowledge of viral sequence space and virus–host interactions preclude accurate prediction of their roles and impacts [1]. Bacteriophages, viruses that infect and replicate in bacteria, are the most abundant self-replicating organisms on Earth. Phages outnumber bacteria by 10 to 1 with an estimated global population of 10^31^ [2]. The increase in antibiotic resistance has sparked the development of bacteriophage agents for several applications in agriculture, biotechnology, and medicine [3]. Before we can truly understand how to apply bacteriophage agents, we must first understand the relationship between bacteriophages and their hosts, as well as other species that could potentially be affected.

Methicillin Resistant *Staphylococcus aureus* (MRSA) is one of the major causes of antibiotic resistant clinical infections. Between 1999 and 2005, hospitalizations for *S. aureus* increased from 294570 patients to 477927. Moreover, MRSA was responsible for 127036 patients in 1999 increasing to 278 203 by 2005 [4].

*S. aureus* has a mesh-like cell wall composed of cross-linked polymer peptidoglycans (PG). Penicillin-binding proteins (PBPs), mediate the final stages of PG synthesis [5]. Methicillin is a *β*-lactam antibiotic that inhibits the transpeptidation domain of PBPs, which weakens the cell wall [6]. MRSA produces PBP2A due to the *mecA* gene that encodes it. Furthermore, this *mecA* gene is transducible by prophage [7].

Through transduction, horizontal gene transfer, bacteriophages could cause *Staphylococcus aureus* to become methicillin resistant through the *mecA* gene. A well-studied example of an adaptive trait conferred by transduction by lysogenic phage is the *mecA* gene transduced by the phage *Staphylococcus sciuri* [8]. Transduction of this temperate phage into the *Staphylococcus aureus* genome confers resistance to broad spectrum beta-lactam antibiotics [7].

## Bacteriophages impact host evolution

Temperate bacteriophages, bacteriophages whose genome is incorporated into the host bacterium, can switch between the lytic and lysogenic life cycles [2]. This can be triggered by environmental stressors such as toxic chemicals and low nutrient conditions. The lytic cycle destroys the host, but if the phage stays lysogenic it provides several benefits. One benefit is protection from secondary phage attacks from another prophage. Temperate phages can lose their switching ability if there are mutations in the attachment sites. Changes to the gene that encode the recombinase responsible for the excision of phage can result in ‘grounding’ of the phage [9]. Grounded phage offers the host benefits, without the risk of entering the lytic cycle.

Lysogenic phages are transduced into the host bacterial genome as prophage sequences and can have a range of selectional impacts on the host, spanning the breadth of the mutualism-parasitism continuum [10]. It is hypothesized that prophage sequences that confer a selective advantage to their host are more likely to be conserved in the bacterial genomes than those that are neutral or deleterious to their hosts [11]. The resultant expectation is that prophage sequences will contain an elevated quantity of genes conferring adaptive functions to host bacteria.

### Computational advances for whole genome sequence (WGS) analysis

The number of sequenced and annotated phage genomes is relatively small with 40 981 phage genome sequences, and 266 129 prokaryotic genome sequences [12] on 18 August 2018. Given the exponential increase in the number of genome sequences deposited in public repositories, it is timely to take advantage of these sequences to analyse them for novel functions. In this study we analyse 10 011 *S *. *aureus* genomes downloaded from NCBI in 2018 for prophage sequences and determine their functions. The total number of genome sequences for all organisms numbered 528 859 for one online repository [13]. Advances in computational techniques for the analysis of large data sets have advanced the omics field by enabling researchers to analyse larger datasets at lower costs [14] .

**Fig. 1.**
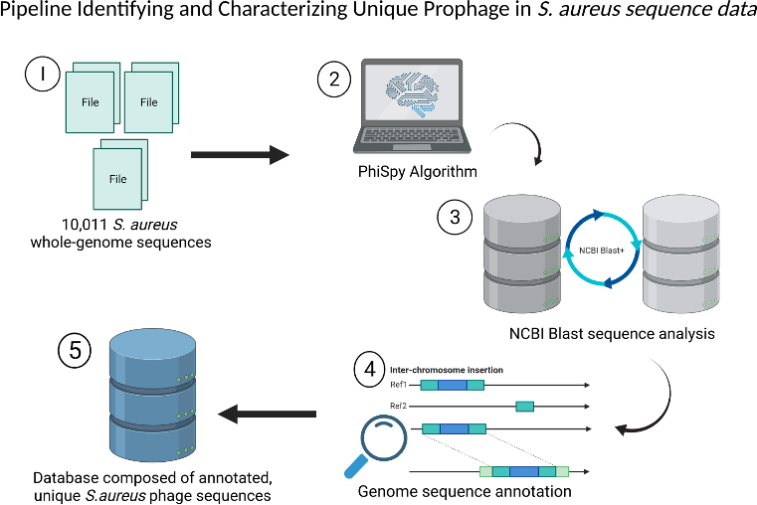
Pipeline identifying and characterizing unique prophage in *S. aureus* sequence data. A visualization of the workflow used to identify unique prophage sequences. 1) 10,011 *S. aureus* genome sequences were downloaded from the National Center for Biotechnology information (NCBI). 2) The sequences were analyzed by PhiSpy. 3) The fasta files for each predicted prophage were compared against each other using NCBI Blast nucleotide alignment tool. Prophage sequences that had 90% similarity along their full length were counted the same. 4) Phage sequences were annotated using two independent methods (VGAS, Prokka). 5) The resulting database of annotated, unique phage sequence allows for the identification of gene function encoded within prophage in *S. aureus *. (See materials and methods section for more information).

In this study, we developed a computational pipeline to detect and analyse prophage sequences in nearly 10 011 *S *. *aureus* genomes. To our knowledge, this is the first large-scale application of PhiSpy on a large-scale set of genomes (10011 *S *. *aureus *). We discovered thousands of putative prophage sequences with genes encoding virulence factors and antibiotic resistance. We found genes encoding *mecA*, genes encoding toxins/antitoxins and clusters of prophage sequences that had genes in common. Our results, and methods developed, will facilitate similar studies for other bacterial species and promise to be a useful tool in the study of prophage host evolution. While most genes we identified were known, the clustering and comparison we did for phage based on their gene content is novel. Moreover, the reporting of these genes with the *S. aureus* genomes is novel (Fig. 1).

## Methods

### *S. aureus* genomes

*S. aureus* genomes were obtained from the National Centre for Biotechnology Information NCBI’s Genbank repository on 18 August 2018 [12]. All available genome sequences (*n*=10011 including complete and partial assemblies) were downloaded for this study. The sequences were collected from a variety of backgrounds that include hospital environments, lab strains and animals. (Accession numbers are provided in Supplemental Data).

### Viral detection

Putative prophage sequences were detected using PhiSpy, version 3.2 [15]. PhiSpy uses a random forest algorithm that has been trained on seven distinct features of prophage: protein length, transcription strand directionality, AT and GC skew, the abundance of unique phage words (unique sequence of length 12 base pairs), phage insertion points and the similarity of phage proteins. PhiSpy has 49 available training sets to increase accuracy for specific genomes. We used the *S. aureus* training dataset (option 24) and identified 196727 phage regions in our 10 011 *S *. *aureus* genomes.

### Prophage clustering

Prophage sequences identified by PhiSpy were unique within a genome, but highly redundant between genomes. We identified highly similar prophages between genomes through a reciprocal [16] search. We increased the max_target_seqs to 12 000 (higher than our total number of *S. aureus* genomes) to ensure we captured all possible matches. We also used a custom output format which provided additional information on the alignment.

We then grouped prophages by using an undirected graph approach with nodes of the form: genome *i*, prophage *j*. Edges were added between nodes if they had a blast alignment which exceeded 90 % similarity and 90 % coverage of both source and target based on the Blastn reports. We then identified genomes sharing the same prophage by determining the connected components, resulting in 191 unique phage clusters.

### Cluster validation

Each of the 191 phage clusters were aligned with Muscle v3.8.1551 [17] and ClustalW v2.1 [18] to ensure each phage was similar. A score of 0.0000 indicates that the undirected graph script formed accurate phage clusters.

### Genome annotation

One representative was selected from each of the 191 phage clusters and analysed with two different tools for gene annotation: VGAS [19], and Prokka [20]. VGAS and PROKKA identified ORFS in each of the phage genome sequences. VGAS identifies ORFs through an enhanced version of the ZCurve algorithm [21] that was customized by adding 13 additional identifying variables (45 total) for the classification model, and BLASTP [22] searches for gene prediction. The ORFs were annotated by both tools with default settings. The combination of annotation tools served as a quality check. The genes identified by both tools were manually reviewed and the highest percentage, and the tool that gave the highest number of matches to known databases was selected for the phages annotation. (Annotation reports and accession numbers are provided in Supplemental Data).

### Pairwise sequence analysis

We identified shared genes between phage through a reciprocal blast search using the annotated phage sequences. We constructed a new undirected graph with the nodes being the phage genome and the edges representing genes shared between phages. The output was a .csv file that listed each of the 191 phage with the genes shared with other phages.

### Jaccard index

We used the layout_with_mds option for the layout function of the R package Igraph [23] to visualize the phages with shared genes using the pairwise count matrix for both PROKKA and VGAS. The Jaccard index [24] was calculated using a modified version of the Jaccard index function in R [25] to compare the Prokka and VGAS networks. (See Table 1 in the Results section)

**Table 1.** Jaccard index shows connections between PROKKA and VGAS undirected graphs

Tool	Total amount of genes shared	Shared genes between both tools	Unique shared genes	Highest # of shared genes in cluster	Lowest # of shared genes in cluster
PROKKA	1363	1335	28	73	1
VGAS	1362	1335	27	75	1

This table shows the relationship between phage genomes by their gene content. Specifically, the nodes represent the 191 phage genome sequences, and the edges between nodes indicate the two phages share a gene (as annotated by Prokka and VGAS). We determined that there were 1335 connected components between the 191 unique phage genome sequences. The total number of shared genes between the 191 unique phage sequences ranged from one shared gene to 73 shared genes for PROKKA and one shared gene to 75 shared genes for VGAS (two more edges than the total identified by PROKKA). PROKKA had a total of 1363 connections compared to VGAS 1362. (See *Analysis Shows Shared ORFs between Unique Prophage Sequences* section for more information and Table 1).

### Quality assessment of predicted phage sequences with CheckV

CheckV is an automated pipeline for identifying closed viral genomes, estimating the completeness of genome fragments, and removing flanking host regions from integrated proviruses [26]. CheckV compares to Virus Orthologous Groups (VOGDB), DOE Joint Genome Institute’s IMG/VR, Reference Viral DataBase (RVDB), KEGG Orthology, Pfam A, Pfam B and TIGRFAM databases [26]. CheckV also reports on potential viral and host genes and uses hmmsearch v.3.1b2 and CheckM to determine the quality of the viral sequences [26]. All 191 unique prophage sequences were analysed with CheckV using default settings (see checkv_quality_summary in Supplemental Data).

## Results

**Fig. 2.**
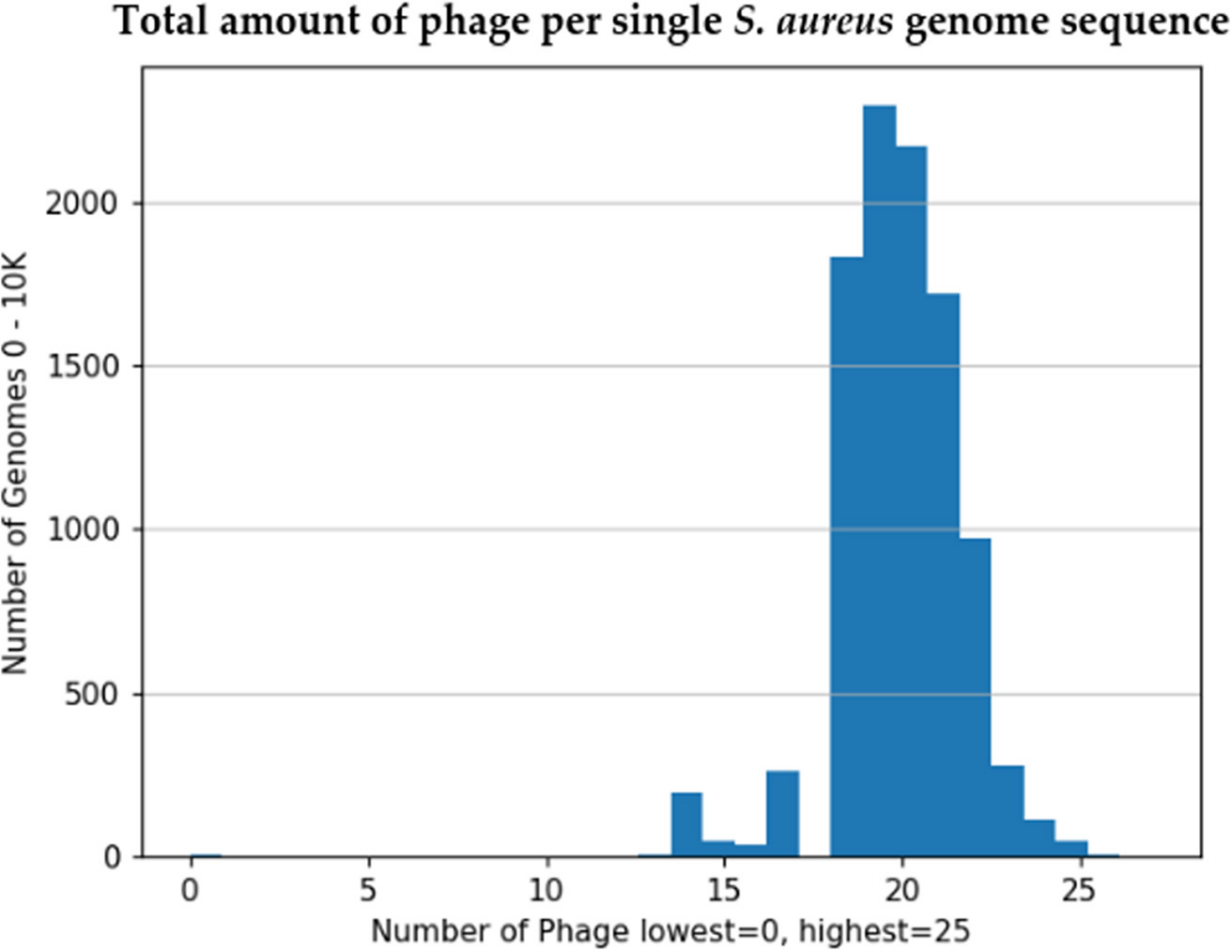
Total amount of phage per single *S. aureus* genome sequence. This figure shows the distribution of the phage genome sequences detected by PhiSpy. A total of 196,727 prophage sequences across the 10,011 *S. aureus* genomes. The x-axis reflects the number of phage sequences per *S. aureus* genome sequence (y-axis). There is an average of 19.68 (standard deviation = 1.78) prophage sequences per *S. aureus* genome. 45 *S. aureus* genome sequences had 25 phage regions present, and 5 *S. aureus* genome sequences had 0 phage sequences detected. (See Analysis Uncovers 191 Unique Prophage Sequences section for more information).

**Fig. 3.**
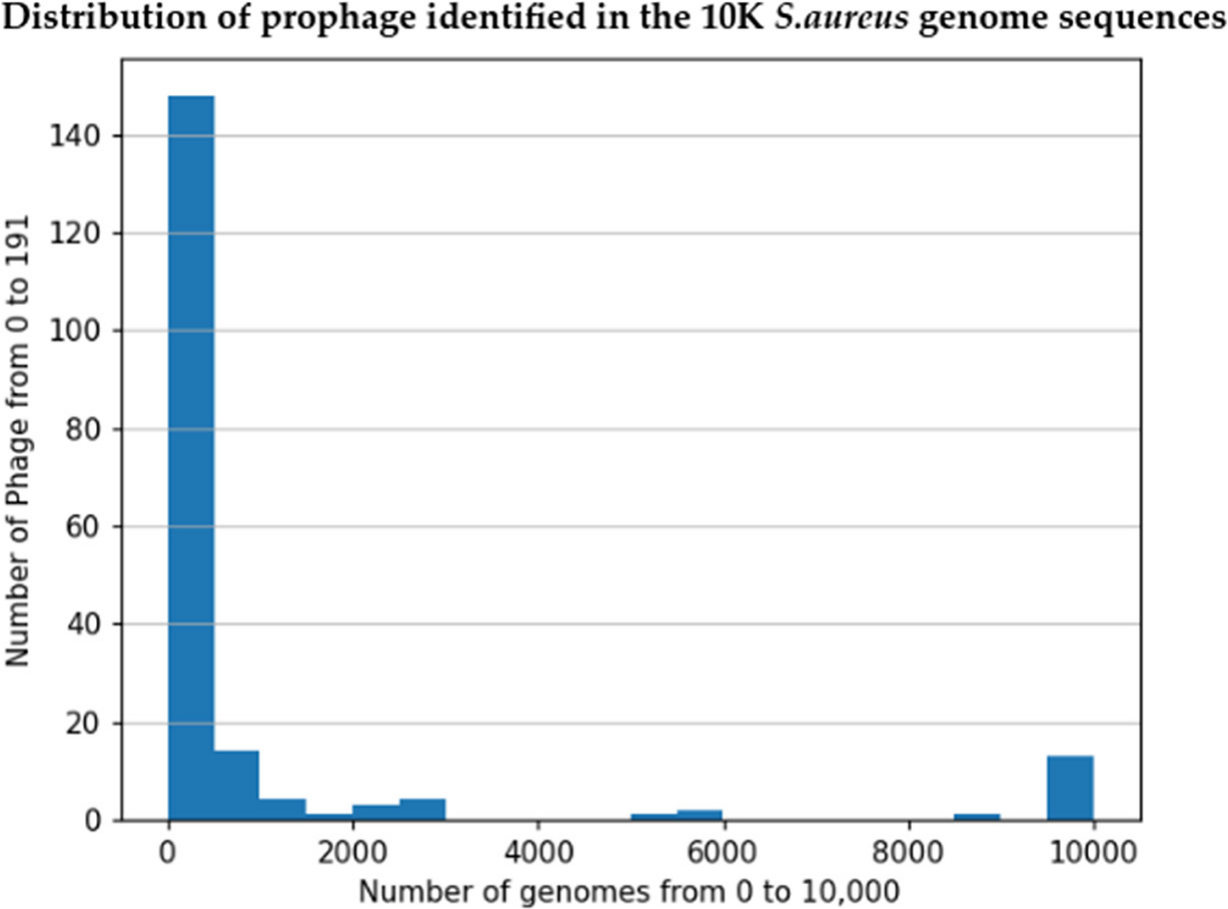
Distribution of prophage identified in the 10000 *S *. *aureus* genome sequences. This figure shows the distribution of 191 unique prophage sequences. PhiSpy detected phage genome sequences in nearly every *S. aureus* genome studied. The detected phage genome sequences were grouped by using an undirected graph approach (see Methods). 1 representative phage from each cluster was selected, totaling 191 unique prophage sequences. The y-axis reflects the exact totals of each of the 191 phage genome sequences that were detected in the *S. aureus* genome sequences (x-axis). (See Analysis Uncovers 191 Unique Prophage Sequences section for more information).

Of the 10 011 genomes initially analysed, 11 were not annotated completely and did not pass the conversion to SEED [27] due to missing locus tags [28]. A further five were too short for PhiSpy to detect phage regions, resulting in a total of 9995 genomes which were used for subsequent analysis. Within these, we detected a total of 196 727 prophage sequences across the 10 011 genomes, with an average of 19.68 (standard deviation=1.78) prophage sequences per genome (Figs 2 and 3).

### Analysis uncovers 191 unique prophage sequences

Reciprocal blast analysis coupled with undirected graph analysis (see Methods) found that the 196 727 prophage sequences corresponded to 191 unique prophage sequences. Each unique prophage sequence appeared in an average of 1024 host genomes (standard deviation=2581.33) (Fig. 3). Each prophage contained an average of 16.83 putative coding regions, resulting in a total of 3207 (VGAS) and 3205 (Prokka) unique open reading frames (ORFs) (Table 2). One phage appeared in all 9995 genome sequences, while 42 of the 191 distinct phages were found in only a single genome sequence.

**Table 2.** PROKKA and VGAS predict gene functions in 191 unique phage sequences

Tool	Total detected ORFS	ORFs with gene function	ORFs with no gene function	ORFs that match known databases
**PROKKA**	3205	2040	45	806
**VGAS**	3207	307	2846	361

PROKKA and VGAS both identified several open reading frames (ORFs). PROKKA determined there were 3205 ORFs for all 191 unique phages, while VGAS determined 3207. VGAS determined that only 307 of the ORFs had gene function, while PROKKA determined 2040 did. PROKKA had roughly 45 ORFS that did not have any gene function identified. This excludes hypothetical or predicted function. (See PROKKA and VGAS reports in Supplemental Data).

### Analysis detects thousands of ORFs with potential gene function

One representative prophage sequence was selected from each of the 191 phage clusters and analysed with two different tools for gene annotation: VGAS [19], and Prokka [20]. VGAS identified 3207 genes, and PROKKA detected 3205 genes (Table 2). For the PROKKA results, 1155 ORFs did not have an identified function. Eight hundred and six predicted ORFs corresponded to known ORFs with accession numbers matching known databases ISfinder [29], NCBI [30], UniProtKB [31]. Two thousand and forty one genes had a predicted gene function. VGAS predicted 2935 ORFs, 361 of which corresponded to known accession numbers matching databases Swissprot and refseq [19, 21] and 307 other predicted ORFs had predicted gene functions. (Table 2).

### Analysis shows shared ORFs between unique prophage sequences

In order to understand how similar the prophage were, for each annotation (PROKKA and VGAS) we created a graph representing genes shared between the distinct prophages. More specifically, the approach outlined in the ‘*prophage clustering*’ section with nodes of the form: genome *i*, identified gene *j*. Edges were added between nodes if they had a matching identified gene. We then compared the edges produced by both tools PROKKA and VGAS with each other.

We found a total of 1335 shared edges defined by PROKKA and VGAS. The lowest number of shared edges between phage sequences was 1, and the highest was 73 (Table 1). There were 1306 shared edges between PROKKA and VGAS, and 28 shared edges unique to PROKKA (Table 1) out of the total 1335 (Table 1). In the 28 unique PROKKA the numbers of shared edges between each node ranged from 1 to 22. VGAS defined a total of 1334 connected components. The lowest number of genes shared between phage sequences was 1, and the highest was 75. There were 27 shared edges unique to VGAS (Table 1) out of the total 1334 (Table 1). The 27 unique VGAS shared edges ranged from 1 to 22 as well.

### Genes encoding *mecA* found in two of the 191 unique prophage

There were several traces of antimicrobial resistance found in the 191 phage clusters. The *mecA* ancestral gene specifically was identified in two sequences. The first sequence, accession number ASM900v1 [12], cluster group has 1023 phage, 10 % of the total *S. aureus* genomes. ASM900v1, or RF122 (ET3-1) provides a framework for the identification of specific factors associated with host specificity in this major human and animal pathogen [32]. RF122 (ET3-1) has several genes involved in host colonization, toxin production, iron metabolism, antibiotic resistance, and gene regulation [33].

ASM323779v1 [34] is the only phage in the cluster, making it individually unique compared to the 196 727 total detected. It is a part of 184 *S *. *aureus* isolates collected from 135 patients over a timespan of 3 years at an Italian paediatric hospital [35].

### Fourty-eight unique gene functions appear in several phage genome sequences

Fourty-eight unique encoding traces of antimicrobial resistance (Shared_genes table in supplemental data). Four genes stuck out the most due to the number of clusters they appeared in. GDAEFEPF_00005 Staphylococcal complement inhibitor, a gene found in ASM2514v1 appeared in ten [36]. GHDFECEE_00007 Superantigen-like protein 13 was found in ASM17451v1 and appeared in eight clusters [37]. ASM17451 also contained GHDFECEE_00008 Superantigen-like protein 13 which appeared in seven clusters. GAIDFPLK_00004 Superantigen-like protein 13 was found in ASM1150v1 and was identified in seven clusters [38].

### Four genes showing traces of toxin/antitoxin (TA) System

Toxin/Antitoxin (TA) systems encode toxin proteins that interfere with vital cellular functions and are counteracted by antitoxins. There are six different types of TA systems [39]. *S. aureus* has genes identified showing types I, II and III [40]. Type I toxin-antitoxin systems have the base-pairing of antitoxin RNA with the toxin mRNA [41]. Type III systems toxic proteins and an RNA antitoxin have a direct iteration where the toxic proteins are neutralized by the RNA gene [42].

Type II, the most studied TA system, has proteic antitoxin that tightly binds and inhibits the activity of a stable toxin [43]. The TA system yoeB-yefM has been detected as genes MBJHDCJA_00021 Toxin YoeB and MBJHDCJA_00022 Antitoxin YefM in ASM900v1 [32, 33]. yoeB inhibits bacterial growth and translation by cleavage of mRNA molecules and is repressed by antitoxin yefM [40]. Enterotoxin Type A causes food poisoning and was identified in three genome sequences [44]. M1022 (NCTC 8325) was identified in two genome sequences [45]. CAFLMJIC_00063 Enterotoxin type A was identified in one genome sequence [32, 33]. (See Shared_genes, Frequent_gene_Functions and Least_Frequent_Gene_Functions tables in supplemental data).

### Thirteen most shared genes in the 191 unique phage

Four genes that stand out the most due to the amount of phage they were found in (Frequent_gene_Functions table in supplemental data). KHDAMHGJ_00009 Chorismate synthase, found in M0471 [45], was identified in 17 phage clusters. Its gene function is shikimate pathway, which shows signs of AMR in plants [46]. EOLKNJBM_00007 Nucleoside diphosphate kinase in ASM1150v1_genomic.gbff_pp18.ffn [38] was found in 16 phage clusters. MIIMDJNA_00002 Heptaprenyl diphosphate synthase component two in ASM24879 [47] was identified in 15 clusters. HGDEFLKI_00006 3-dehydroquinate synthase in M0877_V1_genomic.gbff_pp18.ffn [45] was identified in 14 phage clusters.

### CheckV identifies 63 phages of quality

**Fig. 4.**
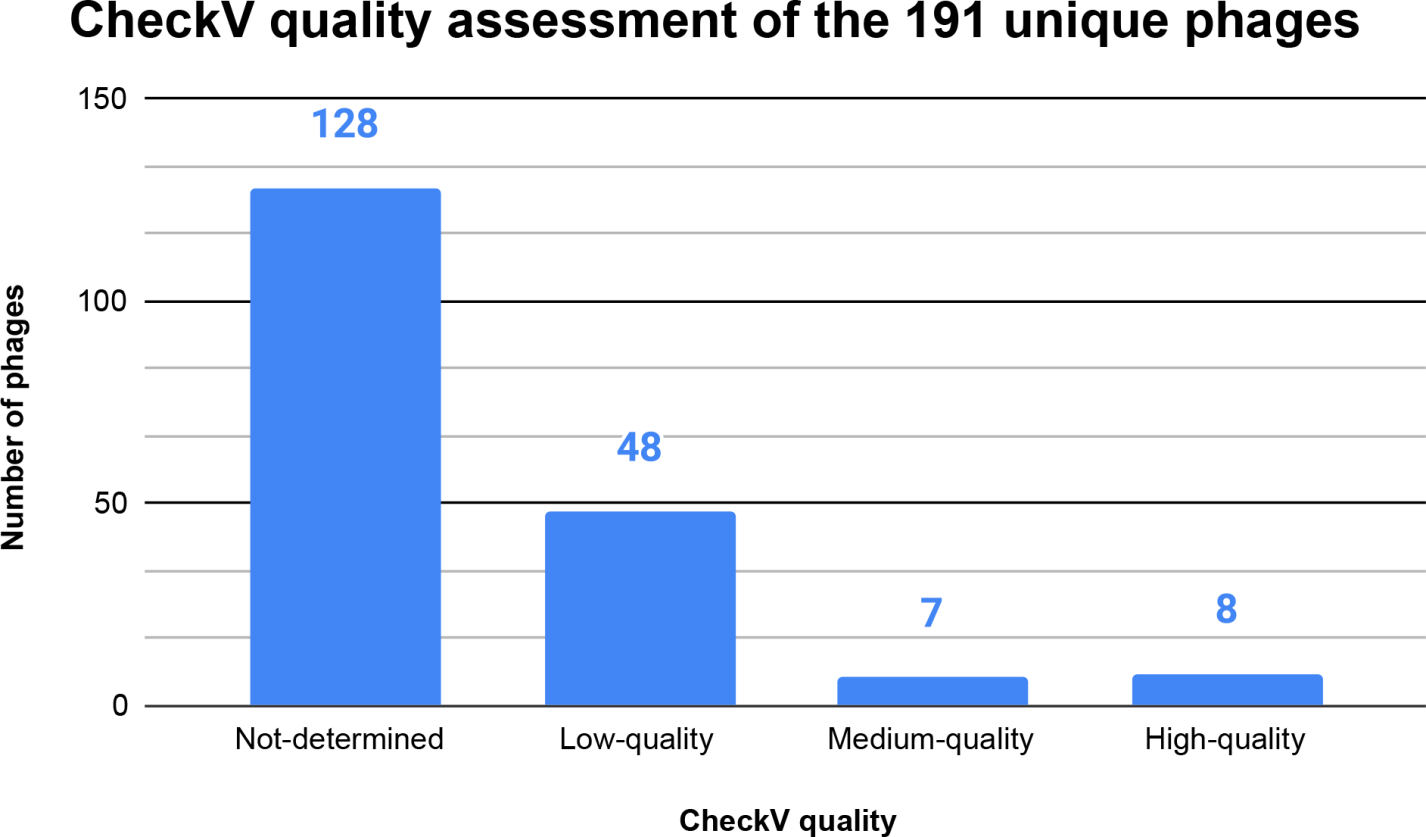
CheckV quality assessment of the 191 unique phage. CheckV determined that 63 phages out of the 191 unique phages were of quality. 48 phages were of low quality, 7 phages were of medium quality and 8 were high quality. The X axis shows the quality of phage determined by checkV. The Y axis shows the number of phages. The totals are shown above each bar. (See CheckV identifies 63 phages of qualityin the results section and PhiSpy_checkv_quality_summary in Supplemental Data).

CheckV analysis determined that there 63 phages that were of quality and 128 that could not be determined (Fig. 4). There were 3277 total genes detected and 310 were viral genes determined by checkV. The high and medium quality phages all had viral genes detected. The low-quality phages had a mix of 23 phages with viral genes detected and 25 without. (See checkv_quality_summary in Supplemental Data).

## Discussion

Determining the presence of virulence and resistance encoding genes in prophage has implications for the potential horizontal transfer of these genes and the functions encode to other bacterial taxa via transduction, and thus can provide insight into the evolution and dissemination of virulence and resistance mechanisms of clinical importance. This knowledge can be useful when creating disease models and novel therapeutics.

The scope of this project is purely computational and determining the functionality of the genes detected would require experimentation. The genome sequences obtained from NCBI may not be representative of the complete diversity of *S. aureus* in nature. *Staphylococcus aureus* subsp. *aureus* strain NCTC 8325 is referenced several times throughout the dataset. It was used as a propagating strain for bacteriophage 47 of the international typing set of bacteriophages and is considered the prototypical strain for most genetic research on *S. aureus* [45]. These limitations need to be considered in the interpretation of our results.

### CheckV analysis identifies 128 potential false positives

**Fig. 5.**
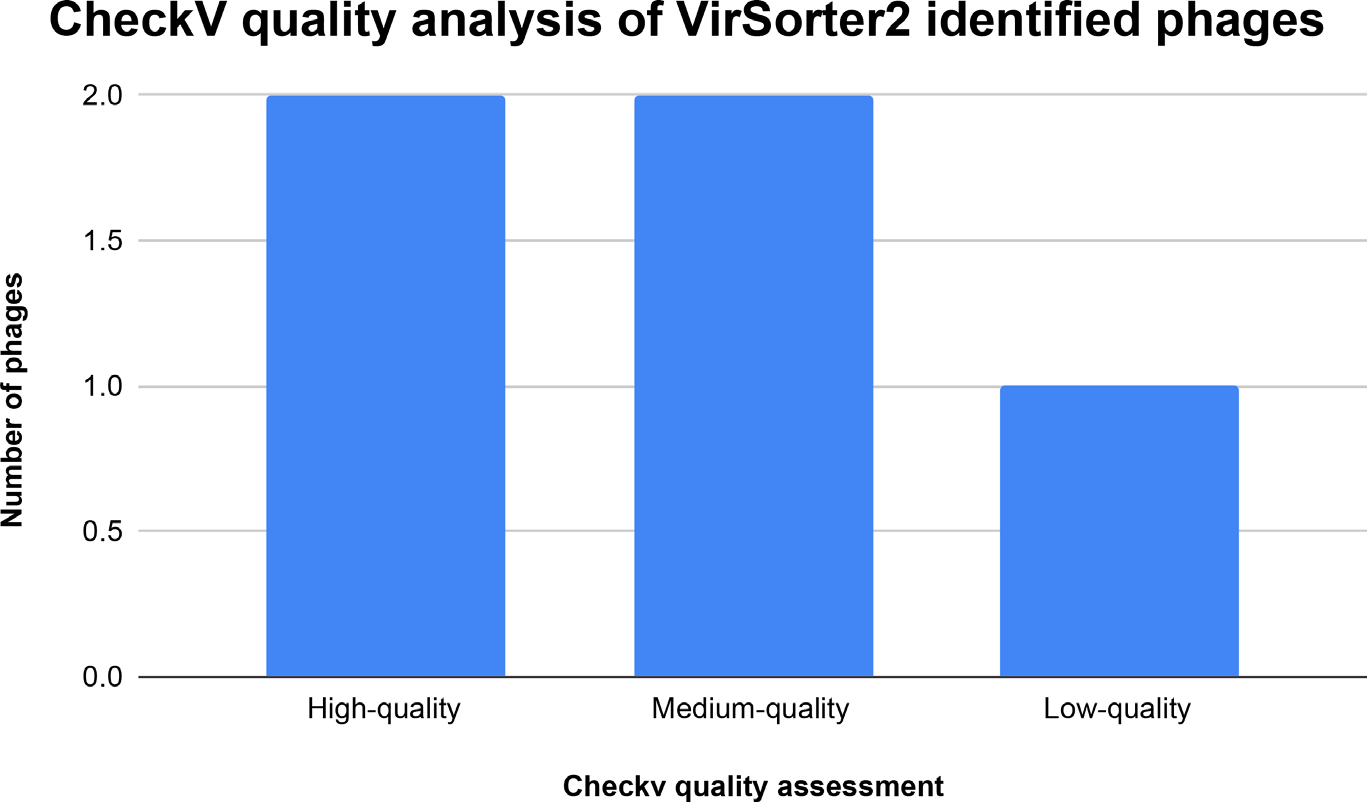
CheckV quality assessment of the virSorter2 identified phage. CheckV determined that all 5 virSorter2 identified phage were of quality. 1 phage was low quality, 2 phages were of medium quality and 2 were high quality. The X axis shows the quality of phage determined by checkV. The Y axis shows the number of phages. (See Analyzing 191 unique phages with virSorter 2 in the results section and virSorter2_checkv_quality_summary in Supplemental Data).

The checkV analysis determined that there 63 phages that were of quality and 128 that could not be determined (Fig. 5), showing that there may be potential false positives. All available *S. aureus* genome sequences were downloaded from NCBI [12] which includes complete genome sequences, and partial sequences or contigs. PhiSpy uses a window size of 40 base pairs and does not rely on known homologues to identify phage regions. The identified prophage sequences appeared multiple times in a *S. aureus* sequence. The combination of PhiSpy identifying the same phages throughout the *S. aureus* sequences that were complete and partial are potentially why so many phages were identified. This is further shown where the 197 727 identified sequences were clustered into 191 unique groups. (See checkv_quality_summary in Supplemental Data).

### Analyzing 191 unique phages with virSorter2

Prophage detection tools have significant problems with false positives and false negatives. PhiSpy identified an average of 20 phages per genome sequence which is a higher number compared to other studies. Deghorain and Van Melderen identified between 1–4 phages per genome [48] and Nepal *et al*. found an average of 3.6 phages per genome [49]. CheckV gave a quality assessment, but further analysis with virSorter2 [50] was done to see if PhiSpy, virSorter2 and CheckV agreed on the high and medium quality phage sequences.

Each of the 191 unique phage sequences were analysed with virSorter2 [50] following a protocol from Guo *et al* [51]. VirSorter2 determined that five of the 191 unique identified phages by PhiSpy [15] were indeed phage sequences. The five virSorter2 [50] identified sequences were analysed with CheckV showing that all five phages were of quality (Fig. 5). The five virSorter2 phages were determined to be quality phage sequences by three different tools showing that the remaining 186 phage sequences were potential false positives identified by PhiSpy [15].

### Databases constrains limit PROKKA and VGAS annotations

There is a large possibility for novel functions to be conferred to bacterial hosts by transduction by lysogenic phage [7]; a significant proportion of the genes encoded by both free living and prophage sequences are of unknown function [52]. There were several virulence factors and toxins identified in the 191 unique prophage representatives, 1 % of the total 196 727 phage detected. This is reflected through VGAS which predicted 2846 genes with no known function, and PROKKA with 45 predicted genes with no known function. PROKKA leverages UniProt [31], RefSeq [53], Pfam [54], and TIGRFAMs [55] databases. VGAS uses RefSeq and SwissProt [56] databases. A third tool MOSGA [57, 58] was used to analyse the 191 unique phage sequences. MOSGA [57, 58] uses EggNog 5 [59], silva [60] and SwissProt [56] databases. Only 34 genes were identified which was lower than both PROKKA and VGAS. PROKKA and VGAS used more databases in combination compared to MOSGA which increases the chances of finding a matching gene function.

Databases that scientists are updating with gene functions from experiments conducted serves a better foundation for gene annotation tools. The databases are limited to what scientists discover in genomics overall and this puts a major constraint on the databases. This could introduce a level of bias in the tools that are using the same databases. (See MOSGA_annotation_analysis in Supplemental Data).

## Conclusion

We developed a novel computational pipeline for phage discovery and annotation and applied this pipeline to approximately 10 000 *S *. *aureus* genomes. In doing so, we discovered 191 unique clusters of putative prophage sequences with genes encoding virulence factors and antibiotic resistance. This computational pipeline consists of first identifying phage genome sequences, grouping them into clusters of identical (or nearly identical) phage, and then identifying genes within these phages. These results will be useful to those interested in bacterial evolution and adaptation, by identifying the mechanism of horizontal transfer of genes that confer adaptive traits to bacteria, especially in the context of antibiotic resistance like the *mecA* gene found in two out of 191 unique phage clusters. This database and pipeline can help guide future experiments by identifying phages and genes of interest.

The immediate next step is to expand the computational pipeline to leverage more tools for phage identification, gene annotation and to show the relationship between phage genome sequences using gene co-occurrence networks [61]. *S. aureus* genome sequences will be collected from the National Centre for BioTechnology Information genbank [62], JGI IMG/M [63], the DNA Data Bank of Japan [64] and phage repositories: ViruSite [65] and inphared [66] to gather more diverse *S. aureus* and *S. aureus* phage sequences. Ultimately the goal is to identify quality phage sequences computationally, and to find and test each identified phage to see if any could potentially turn lytic.

## Supplementary Data

Supplementary material 1
